# Comparability of reference-based and reference-free transcriptome analysis approaches at the gene expression level

**DOI:** 10.1186/s12859-021-04226-0

**Published:** 2021-10-21

**Authors:** Sung-Gwon Lee, Dokyun Na, Chungoo Park

**Affiliations:** 1 School of Biological Sciences and Technology, Chonnam National University, Gwangju, 61186 Republic of Korea; 2 Department of Biomedical Engineering, Chung-Ang University, Seoul, 06974 Republic of Korea

**Keywords:** Transcriptome analysis, RNA-seq, Reference-based assembly, Reference-free assembly, Quantification of gene expression

## Abstract

**Background:**

Lately, high-throughput RNA sequencing has been extensively used to elucidate the transcriptome landscape and dynamics of cell types of different species. In particular, for most non-model organisms lacking complete reference genomes with high-quality annotation of genetic information, reference-free (RF) de novo transcriptome analyses, rather than reference-based (RB) approaches, are widely used, and RF analyses have substantially contributed toward understanding the mechanisms regulating key biological processes and functions. To date, numerous bioinformatics studies have been conducted for assessing the workflow, production rate, and completeness of transcriptome assemblies within and between RF and RB datasets. However, the degree of consistency and variability of results obtained by analyzing gene expression levels through these two different approaches have not been adequately documented.

**Results:**

In the present study, we evaluated the differences in expression profiles obtained with RF and RB approaches and revealed that the former tends to be satisfactorily replaced by the latter with respect to transcriptome repertoires, as well as from a gene expression quantification perspective. In addition, we urge cautious interpretation of these findings. Several genes that are lowly expressed, have long coding sequences, or belong to large gene families must be validated carefully, whenever gene expression levels are calculated using the RF method.

**Conclusions:**

Our empirical results indicate important contributions toward addressing transcriptome-related biological questions in non-model organisms.

**Supplementary Information:**

The online version contains supplementary material available at 10.1186/s12859-021-04226-0.

## Background

Understanding transcriptome dynamics and their impact on gene expression levels is essential for unveiling gene regulatory mechanisms and interpreting genotypic and phenotypic variations. With the recent advent of high-throughput RNA sequencing (RNA-seq) technologies, researchers have gained a powerful tool for not only investigating the expression profiles at the transcriptional level but also identifying novel and non-coding transcripts [1–3]. To date, several transcriptome analysis methods for RNA-seq data have been developed. Based on whether a reference genome is taken into account, two different approaches have been proposed [4–6]. The reference-based (RB) transcriptome analysis method is based on aligning the sequenced reads to a pre-existing reference genome, followed by assembling overlapping alignments into transcripts. In contrast, the reference-free (RF) de novo transcriptome analysis method allows to directly assemble sequenced reads into transcripts by using high levels of redundancy and overlapping of reads, without using a reference genome.

In recent years, many bioinformatics studies have evaluated the advantages and disadvantages of several tools implementing either the RB or RF transcriptome analysis method and have provided guidance for selecting easy-to-handle, reliable, and objective tools. Currently, there are several distinct types of methodological quality assessment strategies for transcriptome assembly. By using a reference genome, multiple RB approaches have been compared, and it has been found that their performances vary with genome complexity, which may potentially complicate correct alignments due to a certain level of variance that may arise from polymorphisms, intron signals, incomplete annotation, and alternative splicing. Therefore, applying relevant methods effectively for handling both low- and high-complexity regions is required [7]. Without using any reference genomes, Holzer and Martz [8] assessed 10 reference-free methods using 9 RNA-seq datasets from 5 different species. The performance of each method was shown to display species- and data-dependent differences. There is no gold standard tool for achieving the best results for any type of RNA-seq dataset. Intriguingly, it has been suggested that in cases where a well-annotated genome from a closely related species is available, this neighbor genome could be utilized to guide de novo transcriptome assembly, albeit with caution [9, 10]. Finally, comparison of differential gene expression analysis results obtained by the RB or RF method have highlighted that 70–80% of the differentially expressed genes are shared [11–13].

Due to the widespread availability and affordability of high-throughput next-generation sequencing technologies, the genomes of numerous species have been sequenced. However, most non-model species lack a high-quality reference genome, and thus, the number of studies comprising transcriptome characterization by RNA-seq has rapidly increased and is continuously growing, particularly in studies related to genetics and genomics. In these studies, RF is the only method available, and according to previous reports, it can very effectively complement the results of genome-based transcriptome analyses in terms of the transcriptome repertoire [14–18]. Although the fragmented and misassembled transcripts from RNA-seq data with intrinsic methodological issues, including low sequencing accuracy, incomplete gene coverage, and chimerism [6, 19], can negatively affect accurate and reproducible quantification of gene expression levels, to the best of our knowledge, no previous study has provided a comprehensive evaluation of the consistency of expression levels between RF and RB approaches.

In the present study, we evaluated whether gene expression profiles obtained by RF and RB approaches could be generally compared. Using six human RNA-seq datasets, we observed that the RF analysis could predict on average up to 80% of the expressed genes; additionally, there was a significant positive correlation of gene expression levels when compared with those of the RB analysis. Expectedly, owing to the intrinsic methodological issues of the RF method, the overall gene expression levels were underestimated by approximately 30–44%. Here, we revealed that this disparity between gene expression levels obtained by RF and RB methods could partly be attributed to the proportion of genes that were lowly expressed, had long coding sequences (CDSs), or belonged to large gene families.

## Methods

### RNA-seq data collection and preprocessing

In this study, we used RNA-seq data from six different human tissues (the brain, colon, heart, liver, ovary, and testis) collected by Zhu et al. [20], which generated high-quality Illumina sequencing-based transcriptome datasets of two technical replicates. The corresponding raw data deposited in the NCBI SRA database (accession no. SRX1830410, SRX1830402, SRX1830412, SRX1830413, SRX1830414, and SRX1830405) were downloaded. To discard low quality and adaptor sequences, all raw reads were preprocessed by Trimmomatic (v.0.33) [21] using the following parameters: ILLUMINACLIP:TruSeq3-PE-2.fa:2:30:10 LEADING:3 TRAILING:3 SLIDINGWINDOW:4:15 MINLEN:36. On average, 55.7 million trimmed reads were obtained per sample (Additional file 1: Table S1).

### Genome-guided transcriptome assembly for RB method

For genome-guided transcriptome assembly, we had previously built the index of reference genome with all assembled human chromosomes (chromosomes 1–22, X, and Y), including the mitochondrial genome, except for unplaced and unlocalized sequences, using Bowtie (v.2.2.6) [22]. Trimmed reads were aligned to the human reference genome using Tophat (v.2.1.1) [23] and HISAT2 (v.2.1.0) [24] with default parameters. Human reference genome and annotation data (GRCh38) were obtained from the Ensembl genome browser (https://www.ensembl.org).

### De novo transcriptome assembly for RF method

For de novo transcriptome assembly, we used the Trinity (v.2.1.1) tool [15], which is considered one of the best assemblers for full-length transcript data obtained by Illumina sequencing [25]. After assembly, CDSs within assembled transcripts were predicted by TransDecoder (v3.0.0; https://github.com/TransDecoder/TransDecoder/wiki) with homology searches (BLASTP with *E* value < 10^–5^) against the Uniprot/Swiss-Prot database (http://www.uniprot.org) [26]. To obtain high-quality non-redundant CDSs, those encoding < 100 amino acids were removed, and CDSs with more than 99% sequence identity were clustered. Of those, the longest CDSs were subjected to further analysis using CD-HIT (v4.6.5) [27] with the following parameters: identity cutoff -c 0.99 and word length -n 5. Non-redundant CDSs were annotated by performing a BLASTP search against the human proteome database from the Ensembl genome browser (https://www.ensembl.org).

### Gene expression level quantification

To assess the abundance of the assembled transcripts, two different quantification methods were performed separately for each analysis. For the RB transcriptome analysis, using Cufflinks (v.2.2.1) [2], genome-aligned reads were assembled into a parsimonious set of transcripts, and their relative abundances were estimated based on the number of reads that supported each transcript. For the RF transcriptome analysis, trimmed reads were aligned to a database containing all non-redundant CDSs using Bowtie (v.2.2.6) [22], and their relative abundances were estimated with RSEM (v.1.2.26) [28] and Kallisto (v.0.46.2) [29]. For human gene families, we downloaded corresponding annotations from the HUGO Gene Nomenclature Committee (https://www.genenames.org) [30]. We used both fragments per kilobase per million reads mapped (FPKM) and transcripts per million mapped reads (TPM) as a unit of gene expression level, and considered a gene as expressed if its FPKM (or TPM) value was found to be greater than one in one or more samples.

## Results and discussion

### Assembly and statistics of transcriptome data

To assemble and annotate the transcriptome with next-generation sequencing data, we employed two complementary transcriptome analysis methods, and to this end, we processed RNA-seq datasets from six different human tissues. First, using the RB approach, a total of 236 million reads were uniquely mapped to the human reference genome, and 58,073 genic regions were covered by 197,856 transcripts. By integrating human reference genome annotation, 20,393 protein-coding transcripts were identified by using the RB method. Next, using the RF method, a total of 334 million reads were used to assemble a reference transcriptome de novo, and of the 691,562 fragments assembled, 75,208 transcripts were obtained. Finally, by performing a BLAST search against the human proteome database, we identified 16,663 protein-coding transcripts using the RF method.

### Comparison of expression profiles generated by RB and RF methods

**Fig. 1:**
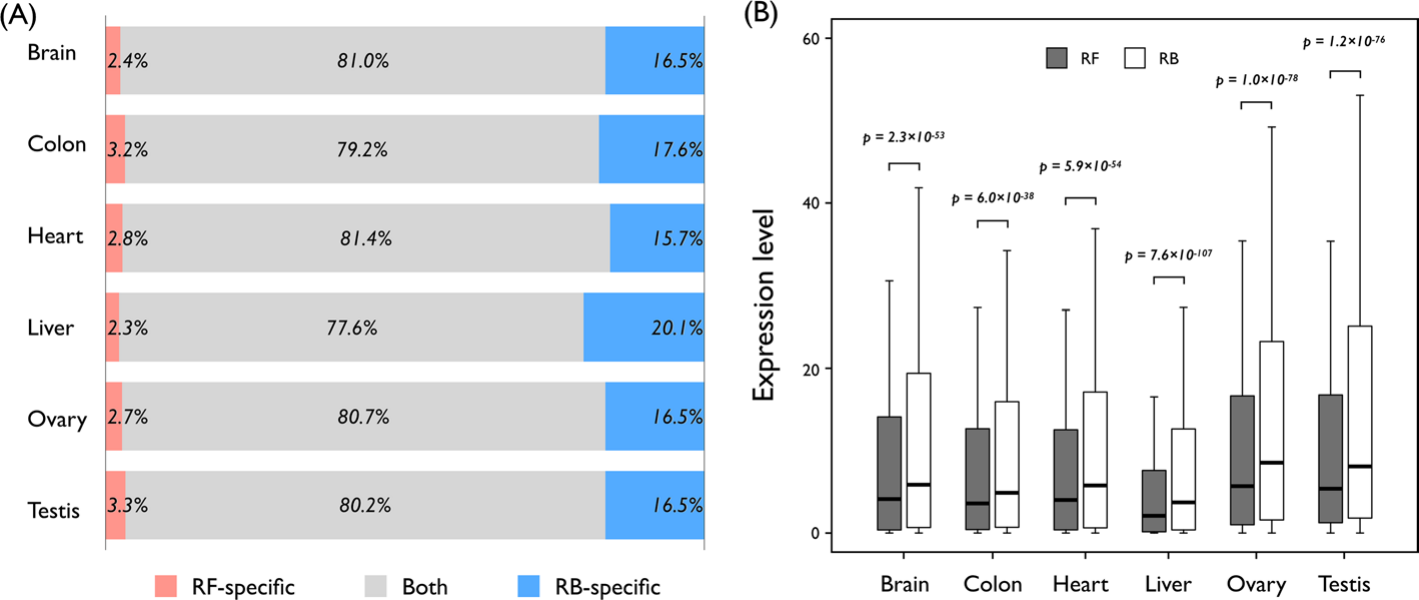
Comparison of transcriptome profiles between reference-free (RF) and reference-based (RB) methods. **a** Proportion of the number of expressed genes identified by RF only, RB only, or both methods. Genes with an FPKM (fragments per kilobase per million reads mapped) value ≥ 1 are annotated as expressed. **b** Expression level differences of commonly identified genes from both RF and RB methods. The central line and lower and upper edges of the box indicate the median and 25th and 75th percentiles, respectively. The whiskers extend to the furthermost point within 1.2 times the interquartile range (IQR). The *p* values shown were calculated using Mann–Whitney *U* test

Previous studies have found that the RB method outperforms the RF method in terms of the transcriptome repertoire [12, 15, 17]. Expectedly, in the present study, most transcripts assembled by the RF method were covered by the results obtained by the RB method, and on average, 17.1% of the transcripts were specific to the RB method. Approximately 80% of the transcripts were identified by both RB and RF methods (Fig. 1a). To examine the quantification consistency of mRNA transcript levels, we compared gene expression levels between RB and RF methods, which were found to be considerably underestimated by the RF method (Fig. 1b). This could easily be explained by fragmented and misassembled transcripts generated by the RF method due to intrinsic methodological issues, including low sequencing accuracy and incomplete gene coverage, possibly leading to less accurate and reliable expression quantification.

**Fig. 2:**
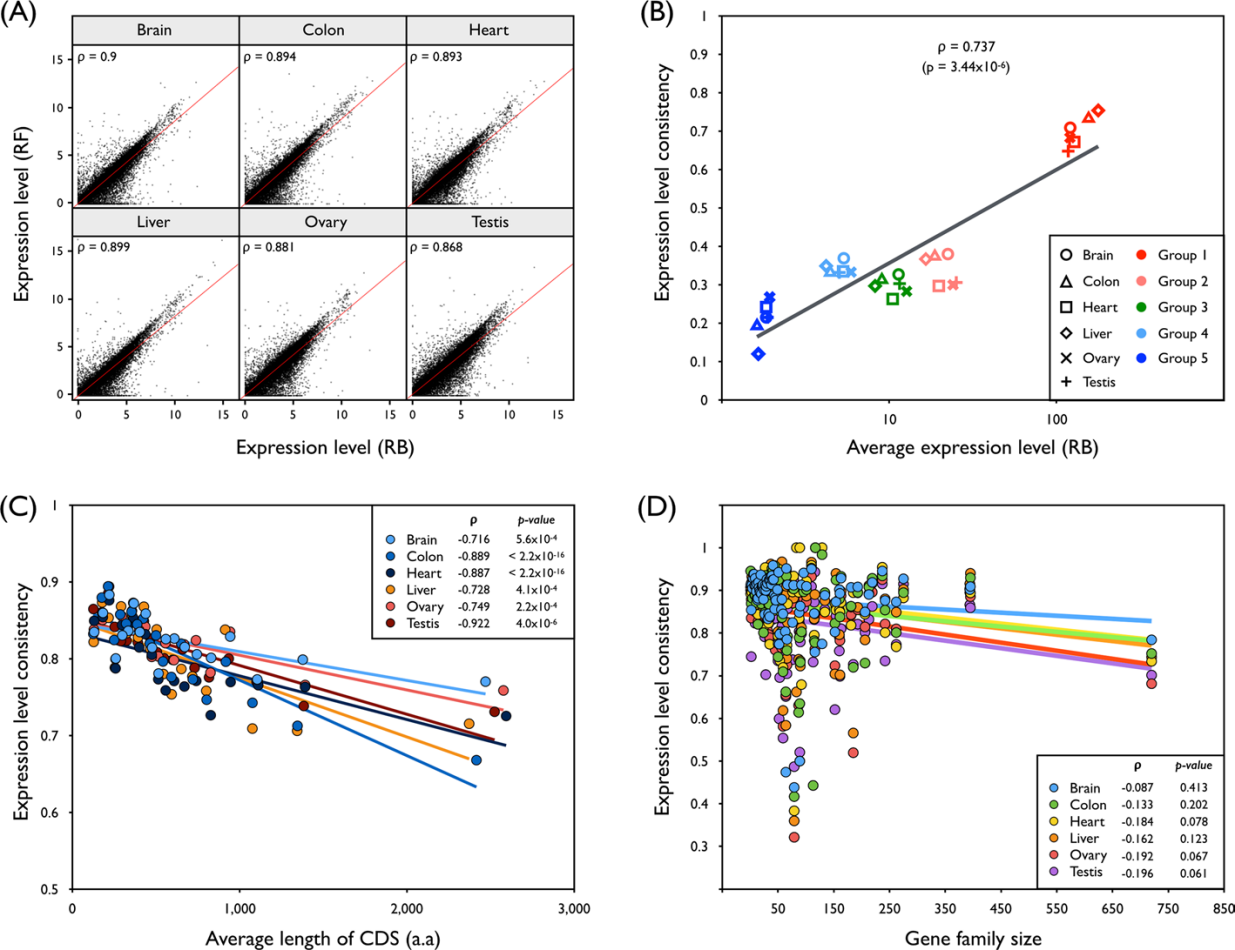
Factors influencing gene expression variability of reference-free (RF) data. **a** Scatter plots showing global gene expression patterns (log_2_-transformed) between RF and reference-based (RB) approaches in different human tissues with linear regression lines (red color). The ρ value indicates Spearman’s correlation coefficient. **b** Correlation between expression level consistency and average expression levels obtained by the RB method. Expressed genes are uniformly divided into five groups with top 0–20% (Group 1), 20–40% (Group 2), 40–60% (Group 3), 60–80% (Group 4), and 80–100% (Group 5) of the expressed genes. **c** Correlation between expression level consistency and average length of the CDS annotated by the reference gene model. **d** Correlation between expression level consistency and gene family size obtained from reference genome sequence data. Solid lines represent the corresponding linear regression. Spearman’s ρ and *p* values were calculated using the cor.test function of the stats package in R

We then investigated which factors could contribute to such expression level discrepancy. The degree of gene expression levels could be one of the potential causative factors for this underestimation by the RF method. To this end, we calculated Spearman’s correlation coefficients (ρ) in advance to explore the consistency of gene expression levels obtained by RB and RF methods (hereafter referred to as expression level consistency) and found a significant and strong positive correlation ranging from 0.868 to 0.9 with *p* values < 2.2 × 10^−16^, suggesting that the RF method could be satisfactorily replaced by the RB method from a genome-wide gene expression quantification perspective (Fig. 2a). However, these high expression level consistencies could be decreased significantly and substantially (ρ = 0.737 and *p* value = 3.44 × 10^–6^) with the extent of the gene expression levels (Fig. 2b). In addition, the extent of the assembled contigs of the transcriptome sequences could be another factor leading to RF method-associated misestimation. Assuming the incomplete and fragmented de novo assemblies generated by the RF method, we observed highly significant, negative correlations between the expression level consistency and length of the CDS (Fig. 2c). Moreover, the existence of gene families, which are sets of genes clustered based on sequence similarities that arose by gene duplication and diversification, can partly explain the expression level discrepancy between RF and RB methods. A large number of paralogous sequence reads from members of the same gene family are often incorrectly de novo assembled, and such newly emerging errors can lead to gene expression quantification distortions. This trend is promoted by an increase in the number of members in a gene family. We examined whether expression level consistency and gene family size were inversely coupled and found a negative correlation in the range of − 0.087 to − 0.196, which was not significant after Bonferroni correction (*p* value < 0.008) (Fig. 2d).

### Robustness of the results

To examine whether the current findings are sensitive to the particular tools or approaches used, we applied the following four alternative tools or approaches. First, instead of using Tophat tool, we used a recently developed widely used RNA-Seq aligner named HISAT2 for the RB method. We found that the comparison results between HISAT2-based RB method and original RF method (Additional file 2: Figure S1) are almost identical to the corresponding comparison results between Tophat-based RB method and original RF method (Fig. 2). Second, instead of using an alignment-based RSEM tool, we used Kallisto, which is based on a pseudoaligment protocol without the need for real alignment, to quantify the abundance of each transcript for the RF method. We compared the results of the original RB method with those of RSEM-based RF (Fig. 2) versus Kallisto-based RF (Additional file 3: Figure S2) methods and found that there are no significant differences. Third, instead of using FPKM, we used TPM value to obtain normalized gene expression levels, and similar comparison results are observed between two quantification methods (Additional file 4: Figure S3). Finally, to investigate whether RB and RF methods are comparable for identification of differentially expressed genes, we calculated fold change of gene expression levels between tissue samples and found significant strong positive correlations in all comparisons (Additional file 5: Figure S4). Together, these results suggest that our conclusion that the RF method could be satisfactorily replaced by the RB method with respect to transcriptome repertoires as well as from a gene expression quantification perspective is robust.

## Conclusions

In the current study, we examined whether the expression level consistency between RF and RB methods was well preserved and found that the RF method could be satisfactorily replaced by the RB method with respect to transcriptome repertoires as well as from a gene expression quantification perspective, together with cautious interpretation of the results. Particularly, when using the RF method to estimate the levels of genes that are lowly expressed, have long CDSs, or belong to large gene families, the results must be evaluated and validated carefully.

## Supplementary Information

**Additional file 1: Table S1.** Statistics of RNA-seq data in six human tissues.

**Additional file 1: Figure S1.** Same as Figure 2, but using HISAT2-based RB method.

**Additional file 1: Figure S2.** Same as Figure 2, but using Kallisto-based RF method.

**Additional file 1: Figure S3.** Same as Figure 2, but using TPM values.

**Additional file 1: Figure S4.** Comparison of fold changes in transcript levels between tissues measured by RF and RB methods.

## Data Availability

All data for this study are publicly available at the NCBI SRA (http://www.ncbi.nlm.nih.gov/sra) website under the following accession numbers: SRX1830410, SRX1830402, SRX1830412, SRX1830413, SRX1830414, and SRX1830405 (Additional file 1: Table S1).

## Abbreviations

BLAST: basic local alignment search tool
CDS: coding sequence
FPKM: fragments per kilobase per million mapped reads
RB: reference-based
RF: reference-free
TPM: transcripts per million
