# Supplementary material for: Comparability of reference-based and reference-free transcriptome analysis approaches at the gene expression level
Source: BMC Bioinformatics. 2021 Oct 21;22(Suppl 11):310. doi: 10.1186/s12859-021-04226-0 (PMC8529712; doi:10.1186/s12859-021-04226-0)
Supplement: Supplementary file 1 — Additional file 1: Table S1. Statistics of RNA-seq data in six human tissues. [file 12859_2021_4226_MOESM1_ESM.docx]

**Table S1. Statistics of RNA-seq data in six human tissues**

| Tissue | Total read bases (bp) | # of raw reads | Read length (bp) | GC(%) | AT(%) | Q20(%) | Q30(%) | # of cleaned reads | % | Accession |
| --- | --- | --- | --- | --- | --- | --- | --- | --- | --- | --- |
| Brain | 6,164,292,398 | 61,032,598 | 101 | 43.87% | 56.13% | 95.94% | 90.53% | 57,430,764 | 94.10% | SRX1830410 |
| Colon | 5,754,163,112 | 56,971,912 | 101 | 43.86% | 56.14% | 96.89% | 92.32% | 53,711,156 | 94.28% | SRX1830402 |
| Heart | 6,711,926,114 | 66,454,714 | 101 | 41.40% | 58.60% | 96.23% | 91.12% | 61,160,848 | 92.03% | SRX1830412 |
| Liver | 5,266,202,620 | 52,140,620 | 101 | 40.21% | 59.79% | 97.02% | 92.63% | 48,074,128 | 92.20% | SRX1830413 |
| Ovary | 6,499,700,470 | 64,353,470 | 101 | 40.88% | 59.12% | 96.04% | 90.81% | 59,294,700 | 92.14% | SRX1830414 |
| Testis | 5,900,540,594 | 58,421,194 | 101 | 42.14% | 57.86% | 96.66% | 92.31% | 54,664,904 | 93.57% | SRX1830405 |
