# Supplementary figures and images for: Comparability of reference-based and reference-free transcriptome analysis approaches at the gene expression level
Source: BMC Bioinformatics. 2021 Oct 21;22(Suppl 11):310. doi: 10.1186/s12859-021-04226-0 (PMC8529712; doi:10.1186/s12859-021-04226-0)

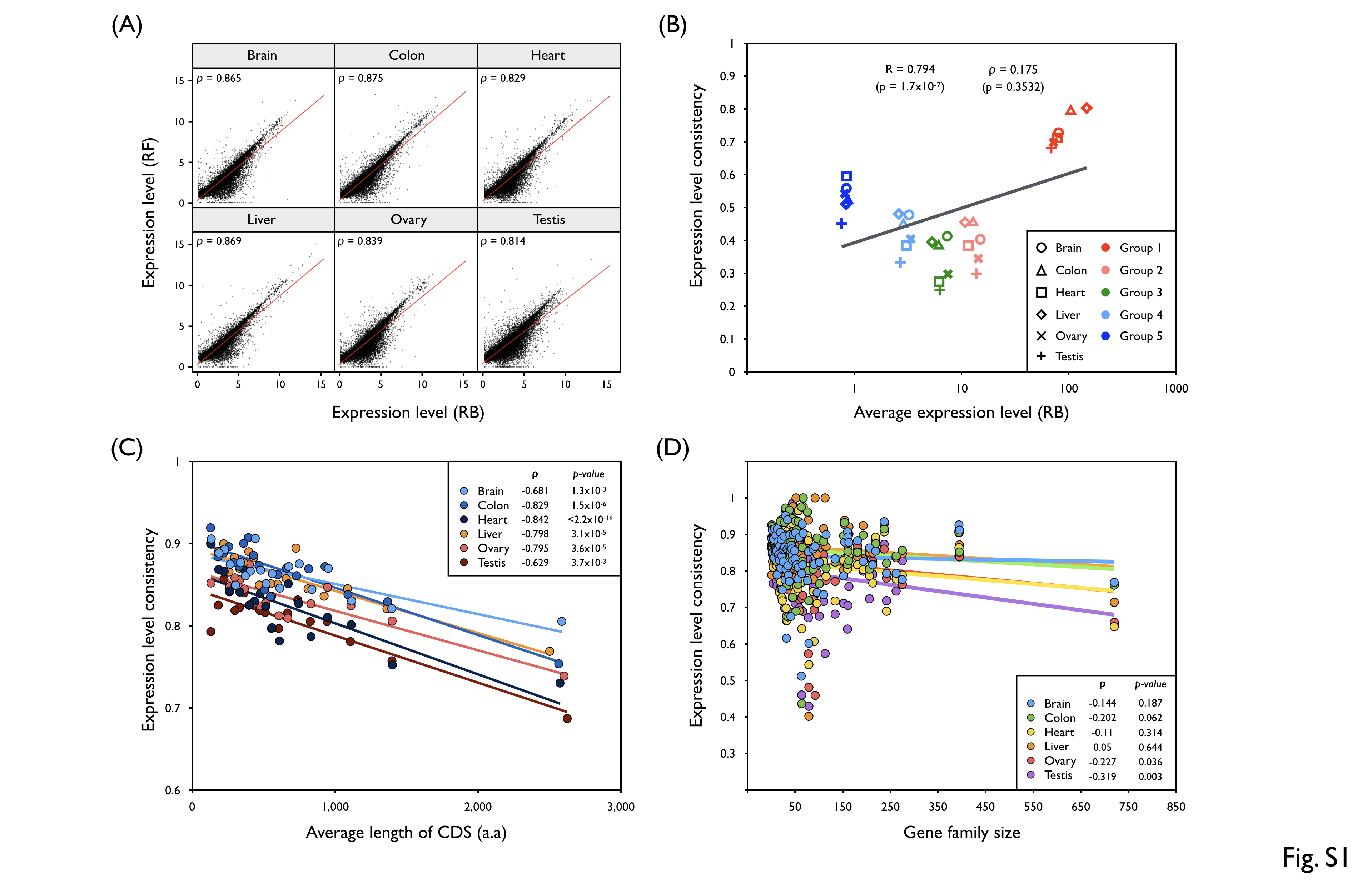

Supplement: Supplementary file 2 — Additional file 1: Figure S1. Same as Figure 2, but using HISAT2-based RB method. [file 12859_2021_4226_MOESM2_ESM.tiff]

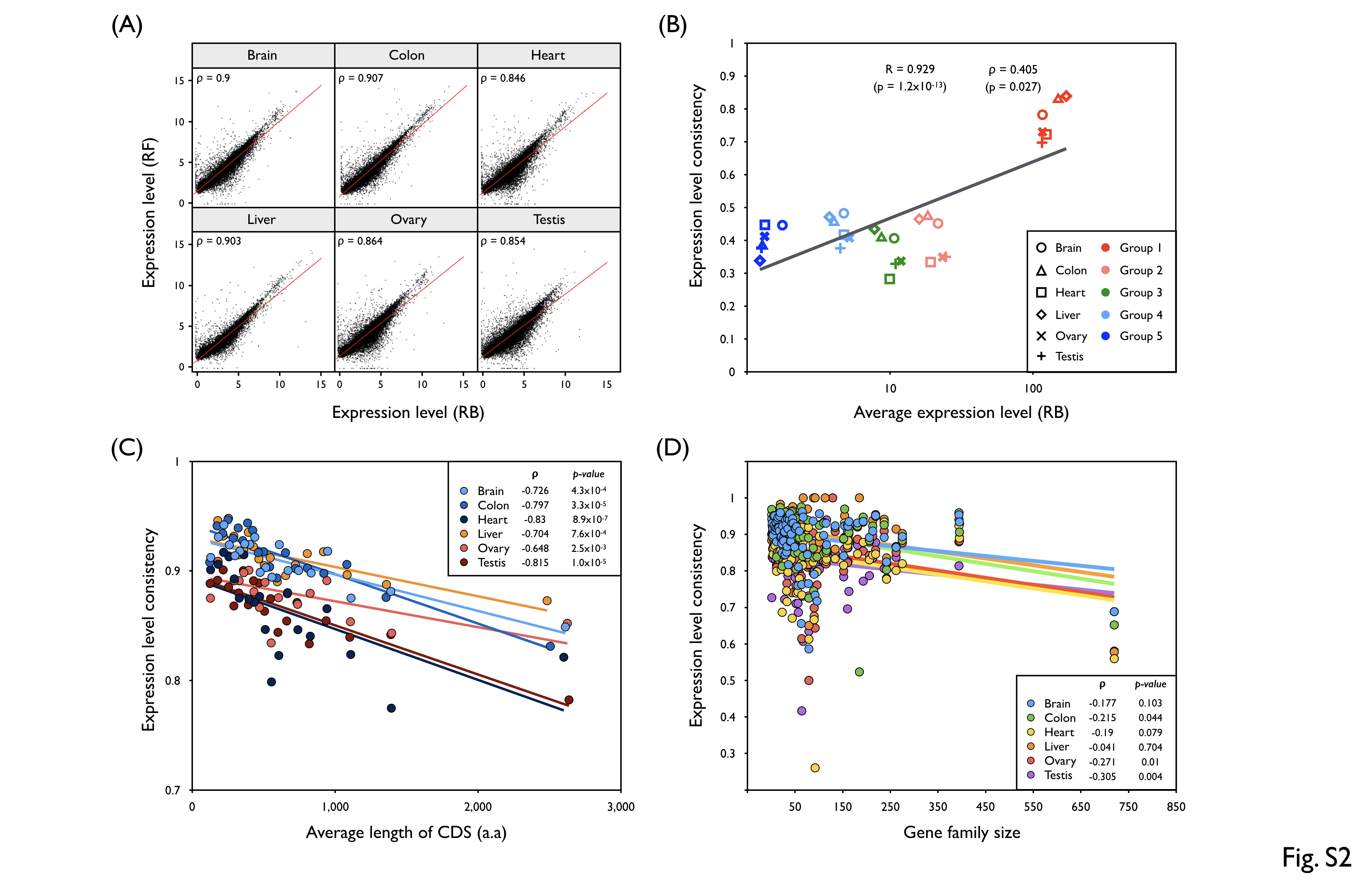

Supplement: Supplementary file 3 — Additional file 1: Figure S2. Same as Figure 2, but using Kallisto-based RF method. [file 12859_2021_4226_MOESM3_ESM.tiff]

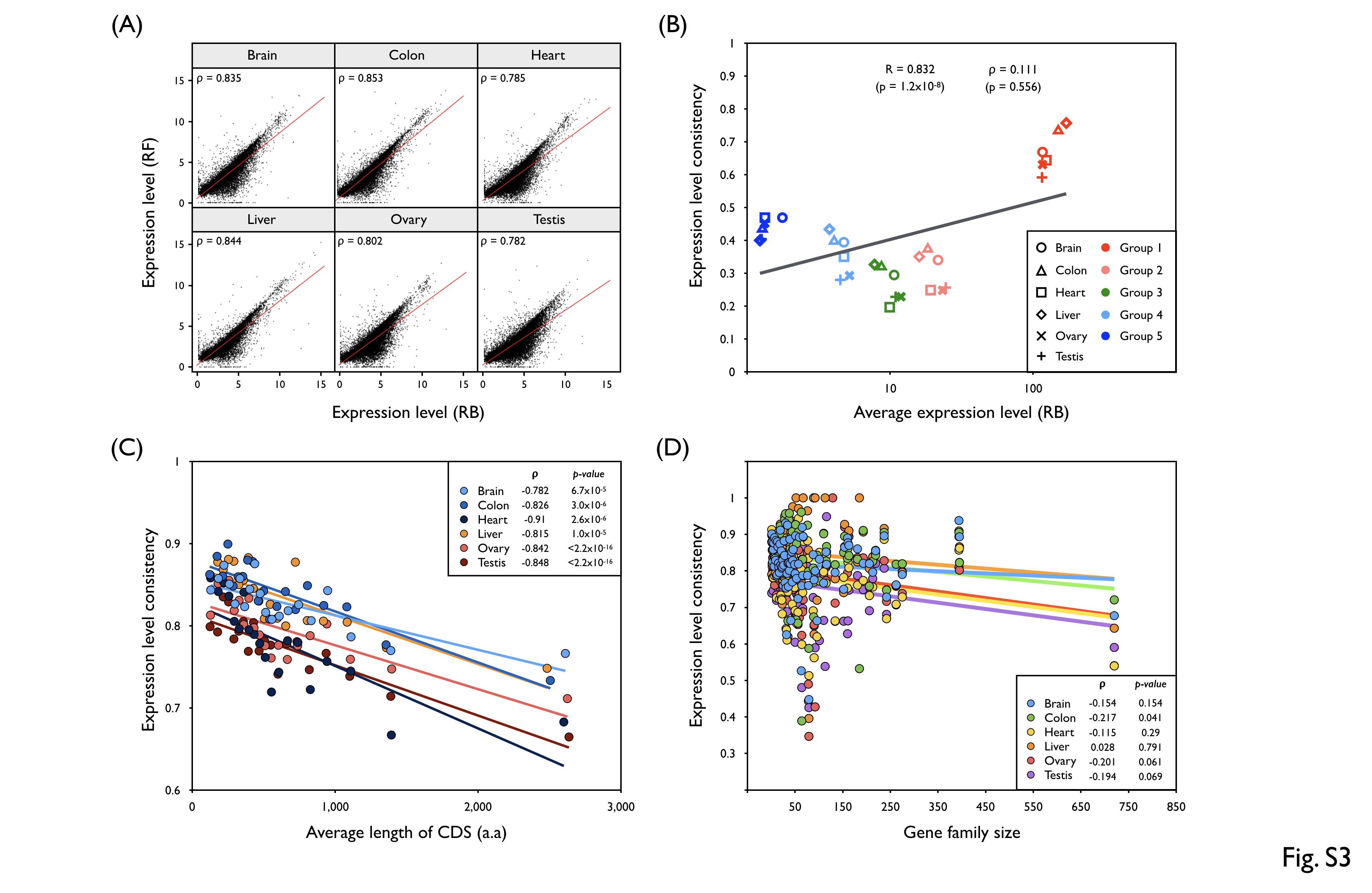

Supplement: Supplementary file 4 — Additional file 1: Figure S3. Same as Figure 2, but using TPM values. [file 12859_2021_4226_MOESM4_ESM.tiff]

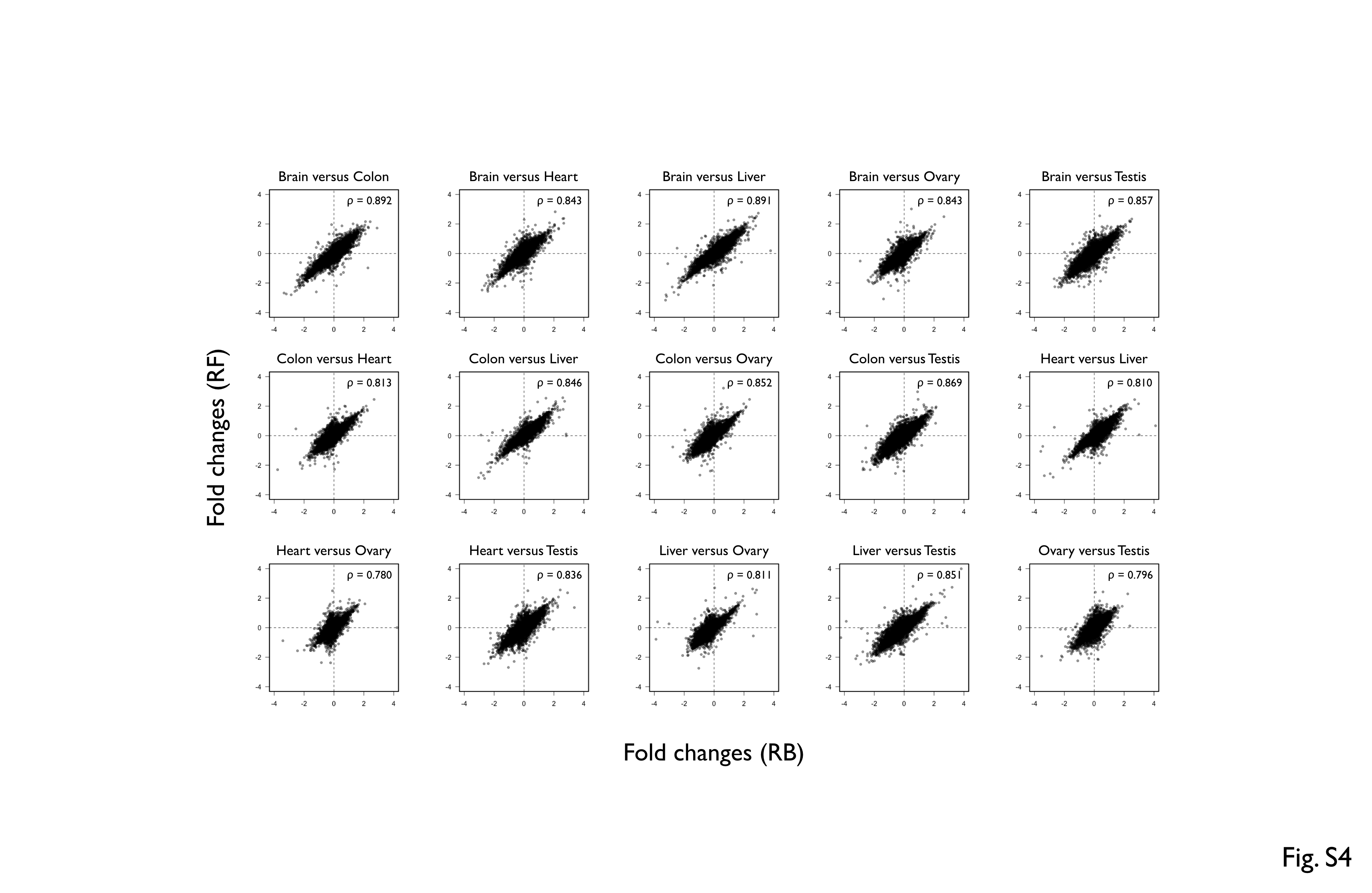

Supplement: Supplementary file 5 — Additional file 1: Figure S4. Comparison of fold changes in transcript levels between tissues measured by RF and RB methods. [file 12859_2021_4226_MOESM5_ESM.tiff]
